# Barriers to Scale of Digital Health Systems for Cancer Care and Control in Last-Mile Settings

**DOI:** 10.1200/JGO.2016.007179

**Published:** 2017-03-21

**Authors:** Shreya Bhatt, Jay Evans, Sanchay Gupta

**Affiliations:** **Shreya Bhatt**, Medic Mobile, India; **Jay Evans**, Medic Mobile, University of Edinburgh, Edinburgh, UK, and Nepal; and **Sanchay Gupta**, Medic Mobile, San Francisco, CA.

## Abstract

The growing cancer epidemic is a major public health challenge globally but especially in low- and middle-income countries where patients often have to make long and complex journeys to receive care. Extending cancer prevention, diagnosis, and treatment to low- and middle-income countries through innovative solutions such as digital health systems is an urgent health priority. We contend that such digital systems will achieve success and scale only when existing gaps in cancer care and control policies and practices are addressed to strengthen health systems and improve outcomes. We call for concerted action to focus on the effective implementation of cancer care and control policies and practice in last-mile settings to improve pathways to care for people with and at risk for cancer.

Medic Mobile, an international nonprofit technology organization, has implemented mobile health or mHealth interventions for cervical cancer care in India, Tanzania, and Nepal since 2014. By using low-cost technology, such as text messaging on basic phones which are ubiquitous and functional in areas with limited infrastructure, first-line health care workers and nurses at primary care centers in India (Madhya Pradesh, Chhattisgarh, and Tamil Nadu), Nepal (Kailali), and Tanzania (Moshi) are able to track the screening and treatment of women with cervical cancer or precancer and monitor and coordinate care for patients over time.

Health care systems in low- and middle-income countries (LMICs) require extensive coordination of care at and across various levels, including the community level, primary care centers, and secondary and tertiary care levels. Improving cancer outcomes in such settings requires innovative solutions that can ensure effective delivery and coordination of care across these levels to reach even the most disconnected communities. Mobile technology offers a unique opportunity to do this: more than 97% of the global population now lives within reach of a mobile phone signal.^1^ Leveraging this extensive cellular phone coverage, mHealth has the potential to change how health care is delivered in low-resource settings, from improving communication and data collection to providing care coordination, enhancing the performance of health care workers, and improving the management of the medical supply chain, all of which ultimately serve to strengthen health care systems.^2^ Over the last decade, the mHealth landscape has evolved rapidly. Experiences from more than 500 pilot programs implemented globally^3^ show that mHealth has the potential to improve coordination of care for cancer in LMICs particularly by strengthening prevention, screening, early diagnosis, treatment, and palliative care in resource-poor settings.^4^

However, mHealth interventions are complex, and introducing any kind of technology into fragmented health care systems such as those in LMICs often fails to have an impact and may also magnify existing systemic deficiencies.^4^ Although technology holds strong promise for improving cancer outcomes in low-resource settings, it should not be seen as a silver bullet for poorly functioning health care systems in these contexts.^5^ We argue that the success of any technology, such as mHealth or health care practice supported by electronic processes and communication (eHealth) interventions, in improving cancer outcomes is contingent on the presence of strong, well-defined pathways to effective cancer care and control policies and practices in last-mile settings.

In the wake of the noncommunicable disease epidemic, a global call to action for cancer care and control has emerged in LMICs.^6,7^ Several LMICs have formed national cancer plans to address the growing disease burden through evidence-based strategies for prevention, early detection, diagnosis, treatment, and palliation, irrespective of the resource constraints faced in these settings. Countries such as Nepal^8^ and Zambia^9^ have also formed national plans to specifically strengthen the prevention and treatment of cervical cancer. Although these and other initiatives represent a step in the right direction, much work still remains to be done. Several gaps exist in the current pathways to cancer care in low-resource settings that result in long and arduous journeys to health care for patients with cancer. Moreover, these gaps hinder the effective implementation and scale of digital health systems for improving cancer outcomes. We believe it is time for the global cancer community to focus on the last-mile implementation of cancer care and control policies and practices in remote settings to address these real gaps in LMICs.

Evidence from mHealth pilot projects implemented in other health care areas suggest that technology can improve outcomes along the cancer care continuum as well.^4^ For instance, digital health systems can help strengthen early diagnosis and treatment of cancer, but only if a strong community-based and -led education program for cancer prevention is in place to change behaviors, raise awareness about cancer, and carefully communicate risk factors and methods of prevention by using culturally relevant messaging. Cancers such as cervical cancer are still heavily stigmatized in low-resource settings, but awareness-building programs can pave the way for more people to seek preventive services, particularly in communities in which preventive care is not commonly sought. In the absence of such strong prevention programs, cancer screening and treatment programs as well as any technology deployed for supporting these programs will face limited uptake among health care workers and patients and will have little success in improving health outcomes.

Care pathways across the cancer continuum and among screening, diagnosis, and treatment touchpoints in last-mile settings also need to be well-defined for digital health systems to function effectively and achieve their stated goals of improving care coordination and health outcomes. Clear referral protocols specific to the structure of the health care system in each LMIC need to be established between the various levels of cancer care coordination, including the community, primary, secondary, and tertiary care levels, so that health care systems provide more integrated and continuous cancer care to patients over time. Cultural, financial, and other barriers that preclude patients from smoothly navigating between these touch points also need to be addressed to support the implementation and success of digital health systems aimed at strengthening cancer screening, diagnosis, and treatment.

We also believe that policies for resource strengthening need to be implemented now to improve cancer care and control in last-mile settings. Given the acute shortage of doctors in most LMICs,^10^ health care providers at all levels of a health care system, particularly nonphysicians such as first-line health care workers and nurses, need to be trained to provide effective cancer counseling, prevention, screening, and treatment; they also need to be trained on the role of technology in strengthening health care systems and improving health outcomes. As we have seen through our experiences in India, Nepal, and Tanzania, first-line health care workers and nurses are often the first point of contact for last-mile communities and play a crucial role in education, prevention, and early diagnosis of cancer. Equipping the first-line health care workforce with capacity building for implementing and using eHealth and mHealth platforms to prevent, screen, diagnose, and treat cancer is of immediate priority.

As mHealth and eHealth technology continues to play an increasingly important role in the delivery of health care in last-mile settings, LMIC health care systems and cancer care policies need to create an ecosystem within which technology interventions can succeed. LMIC governments, donors, private industry, and others need to collaborate and establish common standards for researching and implementing interoperable and user-friendly technologies to strengthen health care systems. Rather than using stand-alone mHealth or eHealth pilots that cannot scale and have limited impact in specific contexts, governments, private enterprise, and others must adopt an open architecture approach to digital health systems. Moreover, scalable, sustainable, and evidence-based technologies with the greatest impact on cancer outcomes need to be identified and integrated into national cancer plans to enhance cancer care coordination, strengthen health systems, and improve outcomes.

The global cancer community also needs to revisit cancer funding priorities in light of the disproportionate burden of disease in LMICs. Estimates suggest that less than 3% of the global investment in cancer research is spent on research relevant to LMICs.^11^ More of this global investment must be allocated toward effecting real grassroots-level change in LMICs, including training and capacity building for first-line health care cadres, strengthening pathways to cancer care, and embedding cancer care services within existing public health frameworks in last-mile settings to effectively address the growing burden of cancer in these contexts.

Creating a vibrant ecosystem in which eHealth initiatives that support cancer care in LMICs can thrive is not solely dependent on innovations in technology; rather, it is very much a function of ground-level policy and capacity within each country. Extending cancer prevention, diagnosis, and treatment to LMICs is an urgent health priority that we can and must address now. We have ample evidence of the growing burden of cancer in LMICs, and we know that pathways to cancer care are fragmented in most LMICs. We also know what works in terms of effective cancer care and control strategies in low-resource settings. And we know that current funding for cancer care pertaining to LMICs is acutely insufficient. What we need is concerted action and more resources directed toward the effective implementation of cancer care and control policies and practices in last-mile settings of LMICs to leverage the full potential of digital health systems and improve pathways to health care for people with and at risk for cancer. LMICs are the next frontier in cancer care, and the global community, including governments, donors, and civil society, must act decisively to extend cancer prevention, diagnosis, and treatment to last-mile and underserved populations around the world.
